# Electrostatic Repulsive Features of Free-Standing Titanium Dioxide Nanotube-Based Membranes in Biofiltration Applications

**DOI:** 10.1021/acs.langmuir.2c03331

**Published:** 2023-02-14

**Authors:** Bogac Kilicarslan, Melis Sardan Ekiz, Cem Bayram

**Affiliations:** † Department of Nanotechnology and Nanomedicine, Graduate School of Science and Engineering, Hacettepe University, Ankara 06800, Turkey; ‡ Advanced Technologies Application and Research Centre, Hacettepe University, Ankara 06800, Turkey

## Abstract

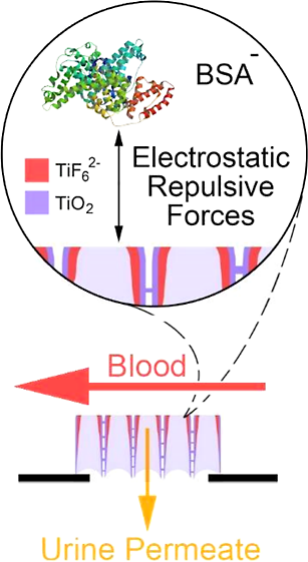

This study presents the electrostatic repulsive features of electrochemically fabricated titanium dioxide nanotube (NT)-based membranes with different surface nanomorphologies in cross-flow biofiltration applications while maintaining a creatinine clearance above 90%. Although membranes exhibit antifouling behavior, their blood protein rejection can still be improved. Due to the electrostatically negative charge of the hexafluorotitanate moiety, the fabricated biocompatible, superhydrophilic, free-standing, and amorphous ceramic nanomembranes showed that about 20% of negatively charged 66 kDa blood albumin was rejected by the membrane with ∼100 nm pores. As the nanomorphology of the membrane was shifted from NTs to nanowires by varying fabrication parameters, pure water flux and bovine serum albumin (BSA) rejection performance were reduced, and the membrane did not lose its antifouling behavior. Herein, nanomembranes with different surface nanomorphologies were fabricated by a multi-step anodic oxidation process and characterized by scanning electron microscopy, atomic force microscopy, water contact angle analysis, X-ray diffraction, and energy-dispersive X-ray spectroscopy. The membrane performance of samples was measured in 3D printed polyethylene terephthalate glycol flow cells replicating implantable artificial kidney models to determine their blood toxin removal and protein loss features. In collected urine mimicking samples, creatinine clearances and BSA rejections were measured by the spectrophotometric Jaffe method and high-performance liquid chromatography.

## Introduction

During the last 15 years, the number of patients struggling with chronic kidney disease (CKD) has increased, although most reasons of CKD, such as genetics and smoking habits, are well known and shared with the community.^1−3^ Unlike acute kidney disease, a definitive treatment has not been found yet for CKD, considering that conventional devices reduce a patient’s daily activities and comfort.^4,5^ Although high-flux hemodialysis machines are extremely efficient in shortening a patient’s hospital retention time by assisting in increasing the reduced glomerular filtration rate (GFR) due to CKD, mechanically stable polymeric membranes inside the dialyzers may cause allergic reactions during operation. Therefore, scientists are still researching to enhance the biocompatibility and antibacterial behavior of polymeric hemodialysis membranes.^6,7^ Synchronously, less allergic, less cloggable, and more biocompatible ceramic membranes and clay mixtures have also been investigated as alternative materials for polymer and polymer-based composites.^8,9^

With increasing progress in the development of nanomaterials and nanotechnology, the volume and weight of the blood filtration systems are drastically reduced, and the patient’s life quality has increased after the emergence of artificial kidneys (AKs).^10−13^ These microblood purification (hemodialysis and/or ultrafiltration) devices reinforce dysfunctional kidneys by maintaining proper GFR without any obstacle to a patient to move independently. The miniaturized devices can be divided into two different categories. Wearable/portable AKs consume dialysate, which is the electrolyte solution used for extracting uremic toxins such as creatinine and urea from blood plasma.^14^ On the other hand, implantable artificial kidneys (IAKs) have no need for any dialysate, pumping system, or power supply because cardiovascular pressure provides the energy necessary for the mechanical diffusion of uremic toxins and water from the circulatory system to the ureter output through the membrane.^15,16^ With the elimination of high pressure driving the filtration through polymeric membranes in conventional hemodialysis devices to sustain the dysfunctional organs, ceramic nanomembranes have emerged in the field with high water permeability in a much smaller surface area.

Nanomembranes used in biological, environmental, and energy applications have been manufactured by various fabrication techniques such as sol–gel, e-beam lithography, nanoimprinting lithography, electrospinning, pressurized/centrifugal gyration, and electrochemical anodic oxidation.^17−26^ Electrospinning, which is one of the methods that has been used for many years, along with the pressurized gyration method, which has contributed significantly to the literature in recent years, are very effective and proven techniques in the mass production of large-size membranes. However, even if the membrane product is a composite, the material to be used in these techniques has to be of polymer origin, and it is not possible to produce pristine ceramic or metal/metal oxide ones. Within aforementioned techniques, it is possible to produce thin polymeric membranes of large size and controllable fiber thickness, but although the fiber orientations can be adjusted, the tortuosity feature of the pores cannot be fully controlled. Electrochemical anodic oxidation is one of the cost- and time-efficient methods to fabricate highly orientated porous or/and nanotubular metal oxides such as titania, alumina, and zirconia surface arrays and membranes, even though it is not a lithographic fabrication technique.^27−29^ Due to their biocompatibility, photocatalytic activity, and antibacterial characteristic, anodic titanium dioxide (TiO_2_) nanotubes (NTs) and their polymeric composites exhibit a reasonable potential in biological applications.^30−35^

The rapidly emerging field of IAKs needs biocompatible and mechanically stable nanoceramic membranes with antifouling ability and high creatinine clearance rate, which can be fabricated with cost and time efficiency. This study shows that electrochemically produced TiO_2_ NT-based membranes have inspiring membrane behavior with high water permeability and creatinine clearance due to low tortuosity and superhydrophilicity of 1D NTs. Due to electrostatic repulsive forces caused by self-formed negatively charged hexafluorotitanate (TiF_6_^2–^) at NT ends, antifouling membrane behavior was observed.

## Experimental Section

Titania NT-based membrane candidates were fabricated by multi-step electrochemical anodic oxidation of titanium foils (125 μm thick, 99.7% purity, Strem Chemicals, USA). Before the anodization process, titanium foils were cut into 25 × 10 mm rectangular sheets. Then, the sheets were cleaned within the ultrasonication bath in detergent, ethanol, and deionized water for 20 min each, and cleansed sheets were dried in an open atmosphere at 20 °C. Electrochemical anodic oxidation was carried out in a cylindrical two-electrode electrochemical cell. The electrolyte solution was prepared with 0.09 M ammonium fluoride (NH_4_F) in deionized water and ethylene glycol with a volume ratio of 2:98. The titanium working electrode and platinum mesh counter electrode were placed in an electrochemical cell at a distance of 20 mm. The multi-step anodization process was divided into three steps. In the first step, anodic oxidation was performed at 60 V for 2 h, and the fabricated primary NT arrays were peeled off with a tape to carve out the nanocavities which led to the secondary NT growth with better orientation. Peeled off samples were cleaned in acetone in an ultrasonication bath for half an hour to uproot residual cohesive substances and then dried in room environment. In the second step, electrochemical anodic oxidation was performed at 60 V for 4 h at a solution temperature of 20 °C in the fresh electrolyte. At the end of second-step anodization, the applied potential was suddenly increased to 120 V and held for 5 min to form three different free-standing NT-based membrane candidates with varying surface morphologies. To define samples, group nomenclature was used. Group 1 was fabricated as the post-anodization temperature was decreased near or below 20 °C for 5 min. Group 2 was fabricated as the post-anodization temperature was decreased near or below 20 °C for 4 min and electrodes were suddenly transferred into a preheated fresh electrolyte at 60 °C, with the same potential applied for a minute. The aforementioned sudden electrode transfer process is schematically described, and the experimental current–voltage–electrolyte temperature graph is given in the Supporting Information (Figure S1). Group 3 was fabricated at the post-anodization temperature of 25 °C. After all post-anodization steps, samples were rinsed in deionized water for few seconds and dried under ambient conditions.

Top and bottom surfaces and cross sections of fabricated titania NT-based membranes were investigated by scanning electron microscopy (SEM). Crystal structures of the pristine titanium electrode, NT array, and free-standing membrane were determined by X-ray diffraction (XRD) analysis. Surface roughness of the open-end surface of NT-based membranes was measured by atomic force microscopy (AFM). Hydrophilicity of the top and bottom surfaces of the synthesized NT-based membrane candidates was characterized by water contact angle (WCA) analysis.

### Pure Water Flux Analysis

A 3D printed polyethylene terephthalate glycol (PETG) pure water flux membrane holder and AK models were manufactured to examine the availability of fabricated nanomembranes for appropriate blood filtration applications. To mount nanoceramic membranes into the 3D printed flow cells, 20 × 20 mm waterproof double-sided tapes with hand-punched holes of 3 mm diameter were stuck between the flow pools of models. Samples were carefully transferred to cover the hole of double-sided tapes. Pure water fluxes of NT-based membrane candidates were determined by crossflow water permeability tests, which is schematically described in the Supporting Information (Figure S2). Pure water feed rate was set as 50 mL min^–1^. After the first 10 min from the beginning, the permeate was collected in a beaker and measured at every 5 min for 1 h.

### Creatinine Clearance and Bovine Serum Albumin Rejection Percentages

To determine biofiltration application performances, creatinine clearance and bovine serum albumin (BSA) rejection percentages were investigated during the cross-flow of blood mimicking solution in 3D printed IAK models. To prepare blood mimicking solution, 0.15 mg mL^–1^ of creatinine and 1.0 mg mL^–1^ of BSA were dissolved in 200 mL of deionized water. Membranes were placed into the module previously described as in pure water permeability tests. The feed rate of the blood mimic driven from the peristaltic pump to the module was set to 50 mL min^–1^. After the blood mimicking solution had been introduced into the test module and the first permeate had been observed, 1 mL of urine mimicking (permeate) samples were collected at the 5th, 15th, 30th, and 60th min of the experiments. The creatinine concentration in samples was measured by the conventional Jaffe method, and the BSA concentration was measured by high-performance liquid chromatography (HPLC). BSA analysis was carried out on an LC-2040C 3D Nexera-i UHPLC system equipped with a photodiode array detector (Shimadzu, Germany). A Thermo Hypersil Gold C18 column (250 mm × 4.00 mm, 5 μm) was employed as the stationary phase, and the gradient elution of acetonitrile and water [containing 0.1% (v/v) trifluoroacetic acid (TFA)] was used as mobile phases A and B, respectively, at a flow rate of 1 mL/min. BSA was first eluted in 20% mobile phase A [0.1% TFA (v/v)] for 2 min, followed by a linear 15 min gradient of 20–60% (v/v) mobile phase A, and ended by 5 min washing/reconditioning in 20% mobile phase A. The column temperature was kept at 37 °C. The calibration curve was obtained by the injection of pure BSA standards repaired in water ranging from 50 to 1000 ppm, and accordingly the chromatograms were extracted at 203 nm to detect the corresponding BSA peak.

### Antifouling Tests

To determine the fouling behavior of fabricated ceramic membranes, the same cross-flow filtration test mechanism was used as previously described in earlier sections. To prepare three different fouling test solutions, 1 mg mL^–1^ of BSA as a fouling agent was dissolved in 100 mL PBS. Acidities of test solutions were set to different pH values by dropwise addition of 0.25 M HCl and 0.25 M NaOH solutions. To ensure complete fouling during tests, acidity of the first solution was set to pH 5.5, and to ensure complete antifouling during tests the basicity of the second solution was set to pH 9.5. For the determination of fouling behavior at physiological alkalinity, the third solution was balanced at pH 7.5 with the aforementioned approach. Before the fouling tests, the solution feed rate was calibrated to 50 mL min^–1^ on the peristaltic pump. After the solution was initialized into the system and the first permeate was observed, the permeating solution was weighed with a high-precision scale for 1 min for each timepoint. The measurements were recorded every 0, 15, 30, 60, 90, and 120 min after the first permeate. After the tests had been continued overnight, samples were collected every 16 and 24 h after the first permeate.

### Cytotoxicity Analysis

The L929 mouse fibroblast cell line was used for the cytotoxicity assay. Cells were thawed from the stock and cultured in the medium consisting of 90% DMEM-F12 and 10% FBS with a supplement of 2 mM l-glutamine in a humidified 5% CO_2_/95% air environment at 37 °C. Meanwhile, a representative titanium dioxide membrane with 2 cm^2^ surface area was immersed in the cell culture medium and incubated for 72 h at 37 °C. L929 cells were harvested at 80% confluency and seeded in 96-well plates at 5000 cells/well and then incubated overnight. The next day, the medium was removed and replaced with the nanomembrane extract and then allowed for another overnight incubation. 10% DMSO in cell culture medium was used as the positive control. The next day, the medium was replaced with 100 μL of fresh medium and 13 μL of MTT solution (1 mg/mL). After 4 h of incubation in the dark at 37 °C, the medium was removed again, and 200 μL of isopropanol-HCl was added to each well to dissolve the blue formazan crystals. After 10 min, the wells were read at 570 nm wavelength, and the percentage of cell viability was calculated. Cell viability was defined as 100% for the MTT assay control, and cell growth was directly proportional to absorbance at a wavelength of 570 nm.

## Results and Discussion

### Fabrication of Titania NT-Based Membrane Candidates

**Figure 1:**
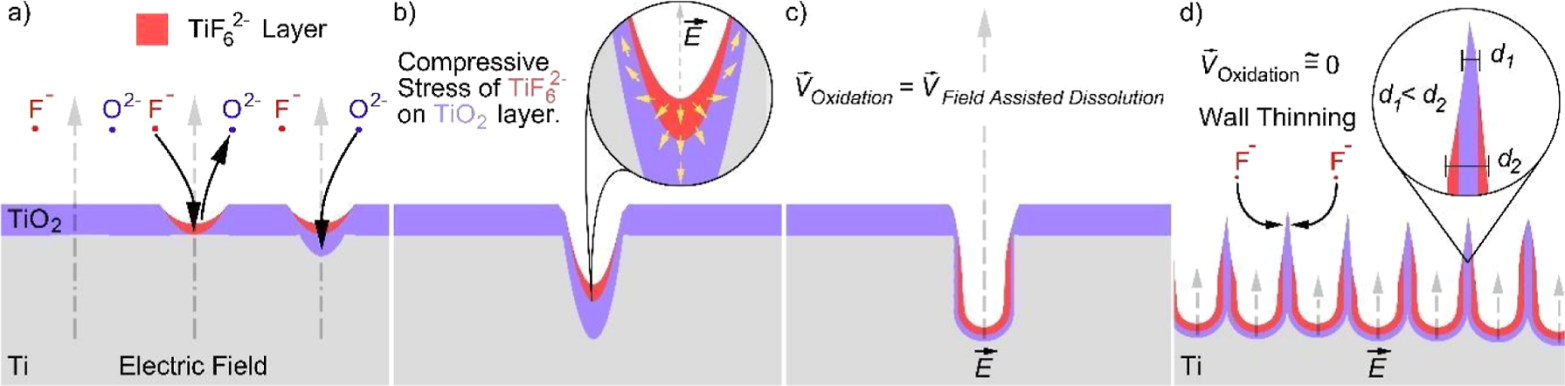
Growth mechanism of TiO_2_ NTs by electrochemical anodic oxidation: dissolution of the primary TiO_2_ layer by F^–^ ions and re-oxidation of the revealed Ti surface by O^2–^ ions under the electric field (a), relief of the compressive stress of the TiF_6_^2–^ layer over the TiO_2_ layer (b), continuous single TiO_2_ NT growth through Ti bulk when field-assisted dissolution rate and oxidation rate are near-equal (c), and wall tapering at the top of NT walls due to the oxidizable Ti layer on the walls during NT array growth (d).

At the beginning of electrochemical anodic oxidation of titanium, the electrode surface starts to be oxidized by oxygen ions (O^2–^) if the electrolyte solution contains any O^2–^ sources such as water (H_2_O) and hydrogen peroxide (H_2_O_2_). The resulting surface chemistry may change depending on the electrolyte composition. If the electrolyte also has fluoride (F^–^) ions that originated from hydrofluoric acid (HF) or ammonium fluoride (NH_4_F), and the formed oxide layer may be dissolved by these ions under the applied electric field, as described in Figure 1a. The hexafluorotitanate transition complex (TiF_6_^2–^) is formed as the end-product of dissolution reaction of TiO_2_. Due to its negative charge, TiF_6_^2–^ compresses the underforming TiO_2_ layer by the electric field direction, as described in Figure 1b. The compressive stress spreads the underforming oxide layer to the outer lower-pressure zones and forms the NTs with different sizes according to the applied potential when oxidation and field-assisted dissolution reactions are isochronous, as described in Figure 1c. NT growth through the Ti bulk happens over the entire surface of the electrode, and hexagonal self-orientation is observed. Chemical reactions that occurred during electrochemical anodic oxidation of Ti are given in eqs 1–5.
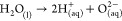
1
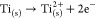
2
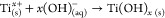
3
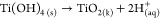
4
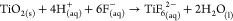
5

At the top of the surface, NT walls start to be tapered by chemical dissolution because of the insufficient oxidizable Ti layer, as described in Figure 1d. As mentioned in most of the relevant studies, the main part of the electrolyte is preferred as non-charged and non-electroreactive monomers such as ethylene glycol, dimethyl sulfoxide etc. to control the NT morphology.^36−39^

**Figure 2:**
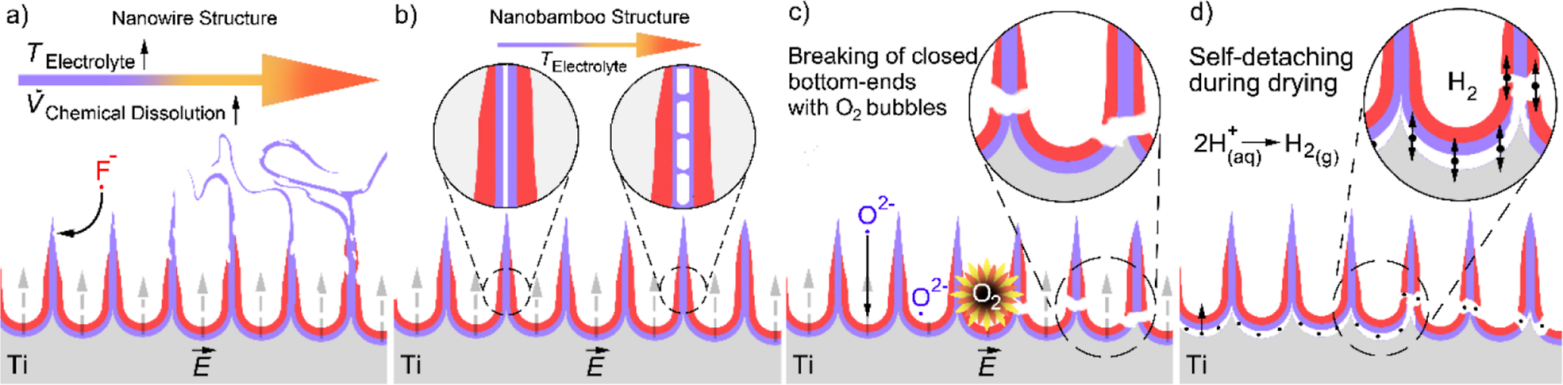
Nanomorphology shift from aligned NTs to chaotic NWs (a) and nanobamboo structure formation between intertubular NT walls (b) due to increasing electrolyte temperature during electrochemical anodic oxidation. Breakdown of closed ends of TiO_2_ NTs due to O_2_ gas bubbling with a sudden increase in applied potential during post-anodization (c) and self-detachment of both open and closed TiO_2_ NT ends from the Ti electrode by H_2_ lifting during drying after anodization (d).

In the fabrication of TiO_2_ NTs with electrochemistry, in addition to the electrolyte concentration and applied potential, the anodization time and electrolyte temperature also affect the NT morphology. A longer application time of the electric field causes deeper/longer NT formation if O^2–^ and F^–^ ion concentrations of the electrolyte are not prominently changed.^40^ The higher electrolyte temperature causes a significant shift of the nanomorphology on the top surface of TiO_2_ NT arrays from NT morphology to nanowire (NW) morphology. This nanomorphology shift is described with increasing kinetic energy of F^–^ ions at the top interior of NTs, and F^–^ ions dissolve tapered walls much faster and randomly crack the thinnest layers of walls without any breaking from their own NTs. Cracked NT walls slowly cover the top surface of NT arrays, as described in Figure 2a.^41^ As the morphology shifts, the nanobamboo structure is also formed depending on the application temperature during anodization, as described in Figure 2b. Increasing temperature can accelerate the diffusion rate of the amorphous oxide layer between intertubular NT walls. These nanobamboo structures also disappear with reduced electrolyte temperatures.^42^

To form open bottom-ended TiO_2_ NT-based membranes, a sudden increase in application potential is one of the most effective and rapid techniques compared to buffered oxide etching of closed NT-ends or lowering of the applied potential. Because the O^2–^ ion speed through the electric field direction is directly related with the applied potential, a sudden increase in the applied potential causes the O^2–^ ions to accumulate at NT ends. Momentarily, accumulated O^2–^ ions at NT ends forming oxygen (O_2_) gas can easily crack NT walls with bubbling, as described in Figure 2c.^43^ During the formation of TiO_2_ NTs under applied potential in the electrochemical cell, the oxide layer at the metal/metal oxide interface is titanium hydroxide [Ti(OH)_4_]. Without an electric field, Ti(OH)_4_ transforms into a more stable oxide form TiO_2_ because field-assisted dissolution is stopped. The liberated H^+^ ions turn into H_2_ gas, and expanding H_2_ gas at the metal/metal oxide interface lifts up the nanoceramic membrane when the sample dries, as described in Figure 2d.^44^ Because electrochemical anodic oxidation causes both electric field-assisted (1D) dissolution and chemical (3D) dissolution of TiO_2_, the end-product TiO_2_ NT-based membranes act as Janus membranes, which are membranes with opposite surfaces having different morphologies, chemical concentrations, wettabilities, and surface charges.^45−48^

### Characterization of Titania NT-Based Membranes

Three different samples with different surface nanomorphologies were successfully fabricated by multi-step electrochemical anodic oxidation. As shown in Table 1, group nomenclature of samples was used to facilitate the comparison between results and surface nanomorphologies caused by slight changes in their post-anodization process.

**Table 1:** Group Nomenclature of Samples according to the Post-anodization Process and Top and Bottom Surface Nanomorphology^a^

	post-anodization process	top surface	bottom surface
group 1	5 min at 18–20 °C	aligned NTs	close-end NTs
group 2	4 min at 18–20 °C	aligned NTs	open-end NTs
	1 min at 60 °C		
group 3	5 min at RT	chaotic NWs	open-end NTs

a NT: nanotubes, NW: nanowires, and RT: room temperature.

**Figure 3:**
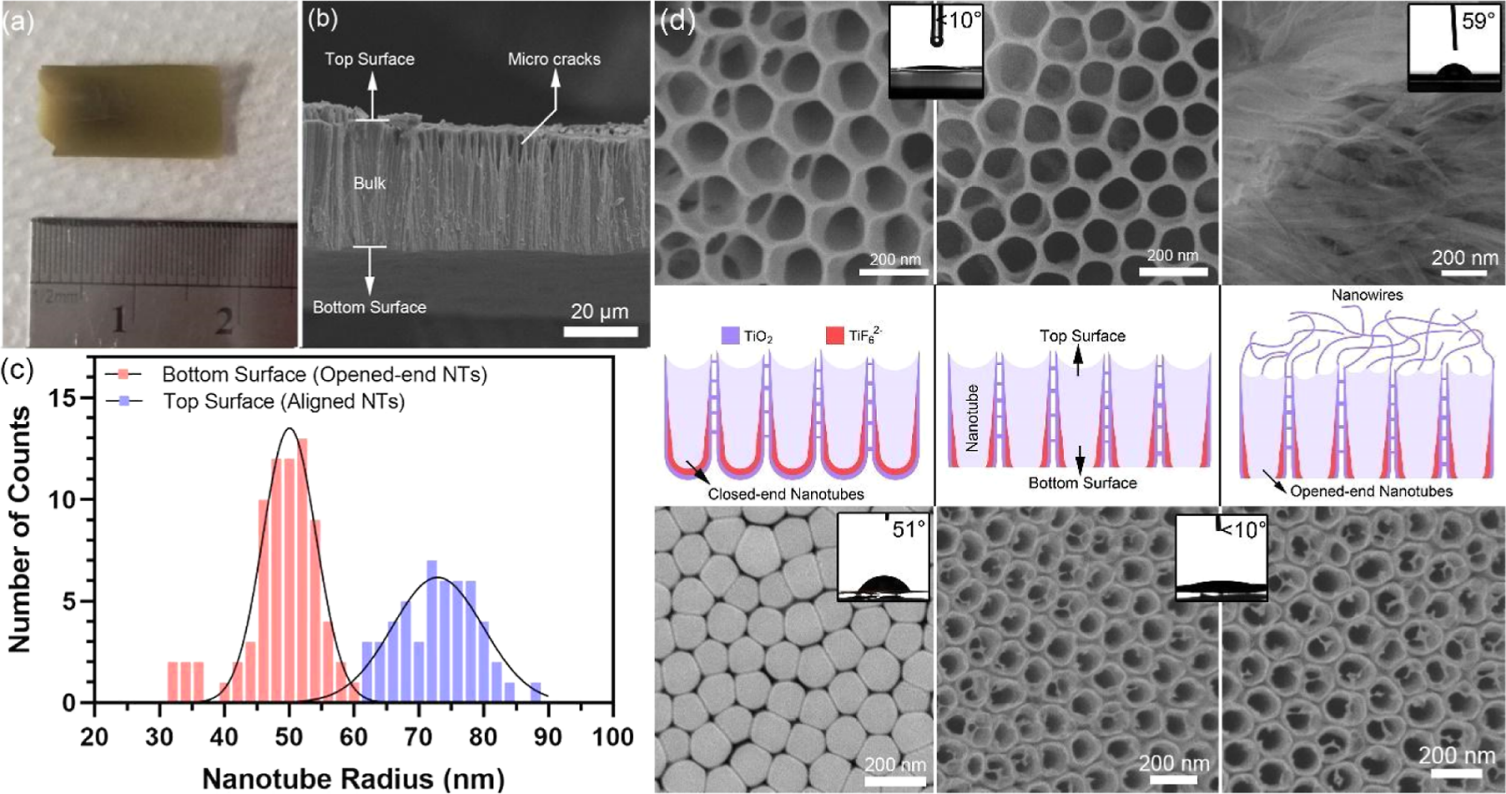
Optical image of the self-detached titania NT-based membrane (a), cross-sectional SEM image of the sample (b), radius distribution on top and bottom NT surfaces (c), and topographical SEM images and WCA results of top and bottom surfaces of TiO_2_ NT-based membrane candidates with schematic expressions (left-to-right in column: group 1–3) (d).

SEM images and WCA results are given with schematic expressions in Figure 3. As shown in the optical image in Figure 3a, self-detachment of the dark-yellow oxide layer as the bulk form was observed in groups 1–3 during drying in an open atmosphere at room temperature after samples were rinsed in deionized water. In the SEM image of the cross section given in Figure 3b, membrane thicknesses were measured to be about 30 μm in groups 1–3. The filtration effective side of the nanomembrane was determined with image processing by comparison of surface pore sizes by ImageJ. The radius of the NTs on the top surface was measured as 72 nm and that of the NTs on the bottom surface was measured as 49 nm, and the size–distribution graph is given in Figure 3c. However, because chaotic NWs were stacked on the measurable pores and close-end NTs are dome-like structures, pore sizes could not be calculated.

In SEM images of the top surfaces, aligned NTs were observed in groups 1 and 2, and chaotic NWs were observed in group 3. In SEM images of the bottom surfaces, close-end NTs were observed in group 1, and open-end NTs were observed in groups 2 and 3. WCA analysis shows that top and bottom surfaces of TiO_2_ NT-based membrane candidates have different hydrophilicities. The surfaces consisting of close-end NTs (51°), and chaotic NWs (59°) were less hydrophilic than the superhydrophilic aligned NT and open-end NT surfaces (<10°).^49^

During the investigation of membrane surfaces for homogeneity with SEM, microcrack formation was observed on the top surface of groups 1–3, as given in the Supporting Information (Figure S3a), although the surface consisting of open-end NTs in groups 2 and 3 had no visible morphological defects such as microcracks or accumulations. These microcracks were also seen in the cross-sectional image of the samples, and the cracks end several micrometers below the top surface, forming narrow valley-like structures. Chemical dissolution at the top of TiO_2_ NTs causes their walls to be thinner, as previously described in Figure 1d, and this morphological surface defect occurred by the accumulation of these tapered upper walls. Additionally, due to tapered NT walls, nanomorphology of the top surface shifts from aligned NTs to chaotic NWs described in detail in earlier sections. Although chaotic NWs completely cover the top surface, narrow valley-like microcrack formations are still present below the surface, as shown in the Supporting Information (Figure S3b). Through the NTs, neighboring NT walls were connected to each other with self-organized nanobamboo structures, as shown in the Supporting Information (Figure S3c).

**Figure 4:**
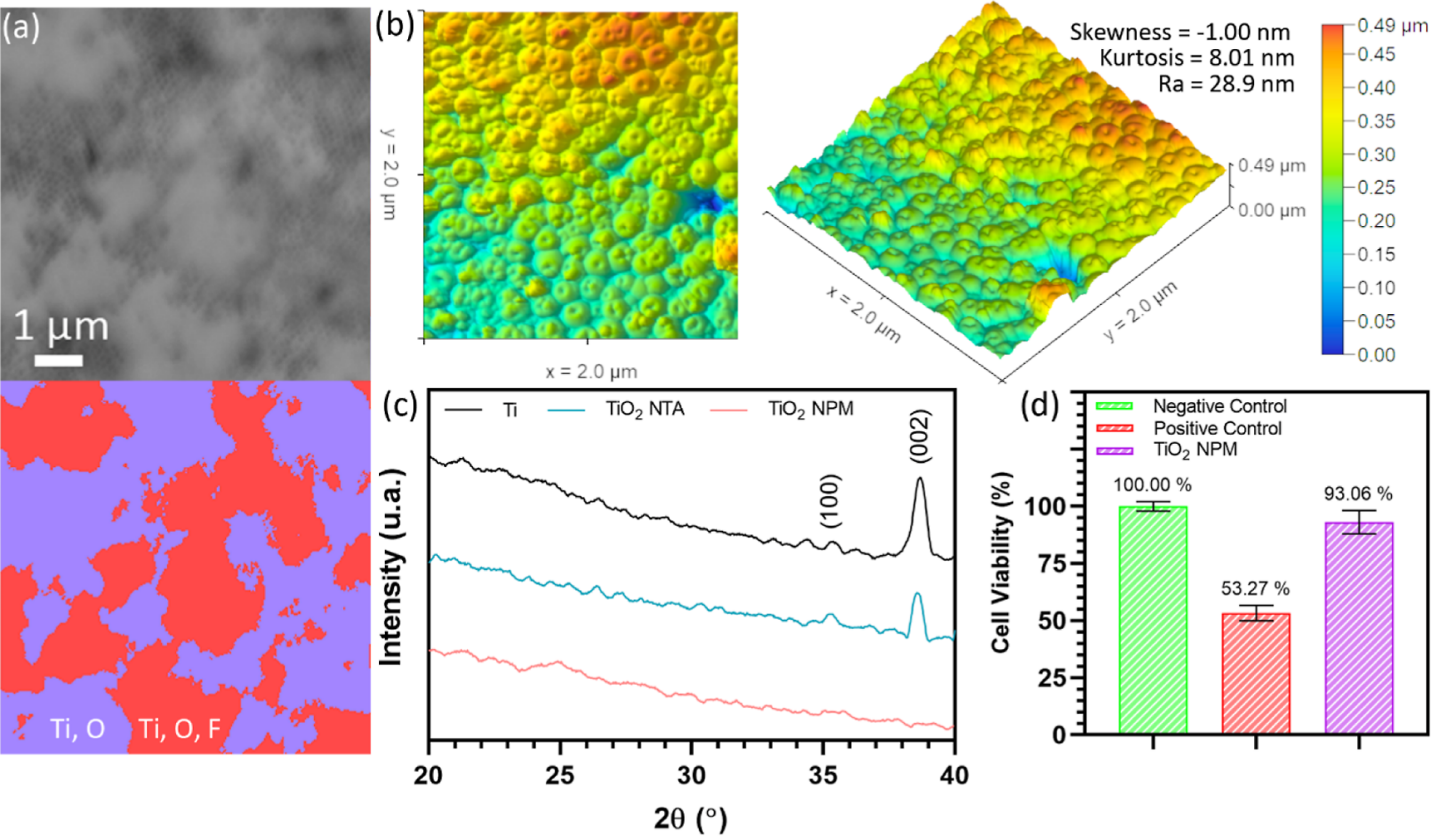
SEM image and elemental mapping of the open-end NT surface according to F signaling Ti and O zones after EDX analysis (a). Topography of the bottom surface of TiO_2_ NT-based membranes collected by AFM (b). XRD graphs of samples in the electrochemical anodic oxidation process: Ti electrode at the beginning (black), TiO_2_ NT array on the Ti electrode surface (blue), TiO_2_ nanoporous membrane after self-detachment (purple) (c), and cell viability percentages of negative, positive, and TiO_2_ NPM after MTT tests (d).

During the energy-dispersive X-ray spectroscopy (EDX) studies, three different atomic signals (Ti, O, and F) were received from the membrane surface used in cross-flow filtration and antifouling tests. As mentioned in the literature, the F signal indicates the negatively charged TiF_6_^2–^ moiety as a remnant after electrochemical anodic oxidation proceeded.^50−52^ In the spectra of 1 and 5 in the Supporting Information Figures S4–S7), the foggy areas of the surface exhibit 17.6% F. In the spectra of 2, 3, and 4, there is no signal of F atoms rather than Ti and O atoms due to some of the surface consisting of not fully opened NT ends. As shown in Figure 4a, some areas of the surface consisting of open-end NTs have a negatively charged TiF_6_^2–^ moiety, which provides the titania membrane an electrostatic repulsive character and antifouling ability.

Since the roughness of a surface interacting with horizontal blood flow is an important factor for hemolysis, this quantity was characterized by AFM. The roughness of the bottom surface of TiO_2_ NT-based membranes was found as 28.9 nm with the non-touch mode. Skewness and kurtosis values are given with surface topography in Figure 4b. In the Supporting Information (Figures S8 and S9), roughness investigation on top surfaces of groups 1 and 3 (clean NTs) and group 2 (chaotic NWs) is also presented. The analysis indicates that the measured surface roughness of opened NT ends is safely below the hemolysis threshold of 0.4–0.6 μm.^53−55^ Although the top surfaces of groups 1 and 3 were also found to be below the hemolysis threshold (210 and 390 nm, respectively), the membranes failed during cross-flow test and was indicated by SEM after the trials, which are presented in later parts of this study. XRD analysis shows that the end-product TiO_2_ NT-based membranes were totally amorphous, although the Ti electrode has (100) and (002) crystal peaks, as shown in Figure 4c. During the fabrication of TiO_2_ NTs, a decrease in intensity was caused by the decrease of the Ti/TiO_2_ NT ratio in the samples which in turn was due to fabricated NTs being in the amorphous form without any heat treatment.^56,57^ MTT assay control presents that the end-product TiO_2_ NT-based membranes have a cell viability of 93.06%. They are biocompatible to be used in biofiltration applications and miniaturized blood purification devices.^58−62^ Normalized MTT test results of control groups and samples are given in Figure 4d.

### Membrane Performance of Titania NT-Based Membranes

#### Pure Water Flux Tests

**Figure 5:**
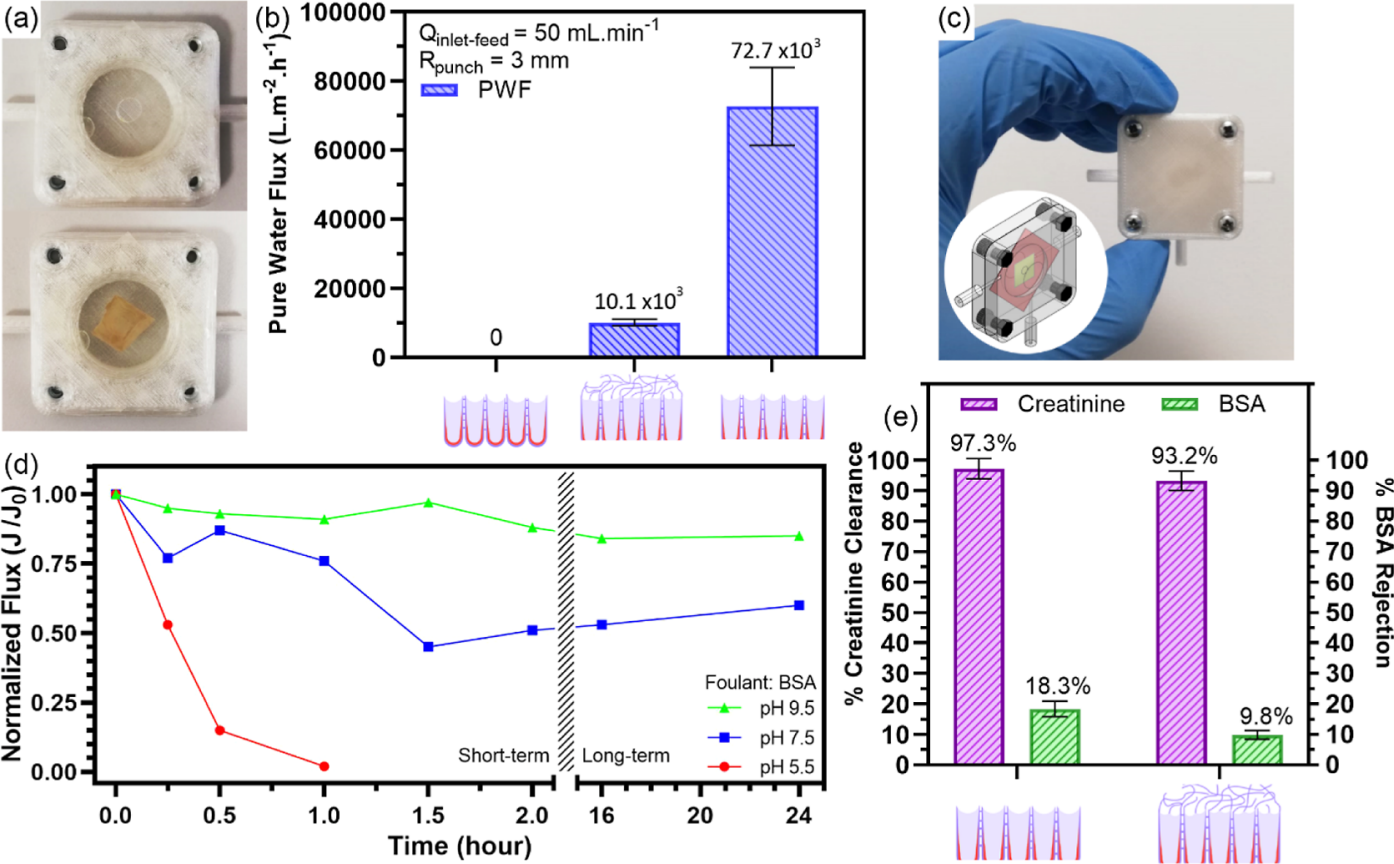
Before and after optical images of membrane placement on a hand-punched double-sided tape (a), comparison of cross-flow pure water fluxes (b), design and optical image of the 3D printed model (c), fouling behavior of titania NT-based membranes for different filtrate acidities (d), and comparison of creatinine clearance and BSA rejection percentages (e).

To compare pure water permeabilities between groups, nanomembranes were carefully placed in the flow cells, as given in Figure 5a. Collected permeate volumes every 5 min for an hour at 50 mL min^–1^ inlet feed rate are tabulated in a graph, Figure 5b. At the end of the cross-flow pure water flux tests, described in the earlier section, the highest permeability was measured in group 2 as 72.7 × 10^3^ L m^–2^ h^–1^ because both surfaces of the membrane exhibited superhydrophilic features as characterized in the previous section. Subsequently, pure water permeability was calculated as 10.1 × 10^3^ L m^–2^ h^–1^ in group 3. The decrease in water permeability is directly related with hydrophilicity decrease of chaotic NWs on the top surface of the nanomembrane.^63−66^ In addition, impermeability of water was observed for group 1 samples by PWF tests, and it strongly proves that group 1 samples do not possess an actual membrane property and intertubular spaces between neighboring NT walls observed in group 1–3 samples do not allow any liquid diffusion or mass transport medium, although the intertubular nanobamboo structure provides the nanomaterial a free-standing behavior.^67−69^

For group 2 and 3 samples, further membrane performance tests had been carried out in the 3D printed PETG IAK model where its design and optical image are given in Figure 5c.

#### Antifouling Tests

To determine the fouling behavior of titania NT-based membranes at physiological alkalinity, the decrease in normalized flux of permeating filtrate solutions at different acidities was investigated. Normalized fluxes were calculated as the ratio of instant permeate flux (*J*) and initializing permeate flux (*J*_0_) as described in eq 6 given below.^70,71^
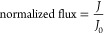
6

In this study, Coulomb’s law was adopted to present the electrostatic interaction between the two same charges (*Q*_1_ and *Q*_2_), where *k*_e_ is Coulomb’s constant and *r* is the distance between charges, as given in eq 7.^72^
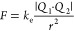
7

In the literature, isoelectric points of titania NT arrays and BSA were about pH 2.4 and pH 4.6, and their negative charges strengthen with higher pH values.^73,74^ Acidities of antifouling test solutions were selected above their isoelectric points to drive on electrostatic repulsive interactions and better understand fouling behavior of samples, and test solutions with pH 5.5, pH 7.5, and pH 9.5 were prepared.

For the solution at pH 5.5, a rapid decrease in normalized flux (*J*/*J*_0_) supports the fact that adsorption of the BSA molecule membrane surface causes the closure of filtration pores. With the cake formation of accumulating BSA on the completely closed pores after 1 h, investigated samples lost their filtration ability with mass transport functionality, which constitutes the definition of a membrane, as expected in a total fouling scenario.^75^ For solution at pH 9.5, a slight reduction in normalized flux after 2 h can be caused by the non-specific adsorption of BSA on the membrane, especially to the zones indicated on the zone EDX results. After 24 h, the stability of normalized flux (*J*/*J*_0_) supports the fact that samples sustain their initial membrane behavior after 24 h, as expected in the total antifouling scenario. For the physiological alkalinity solutions at pH 7.5, normalized flux (*J*/*J*_0_) decreased about 50% after 2 h with potential cake formation of accumulating BSA on uncharged zones. However, due to electrostatic repulsive forces between the charged BSA protein and TiF_6_^2–^-rich zones of the membrane, electrostatic repulsive features are more dominant for the adsorption of proteins. Experimentally observed mass transport that should have been continued through the filtration pores on the negatively charged surface and the stabilization of normalized flux (*J*/*J*_0_) at 50% after 24 h support the fact that titania NT-based membranes have antifouling behavior.

#### Creatinine Clearance and BSA Rejection Tests

To compare creatinine clearance and BSA rejection performances of TiO_2_ NT-based membranes, total percentages of clearance and rejection were calculated from measured concentration divided by initial concentrations for each membrane, as shown in eqs 8 and 9.^14,76,77^
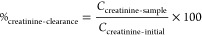
8
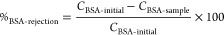
9

For group 2, creatinine clearance and BSA rejection rates were calculated as 97.25 ± 3.28% and 18.33 ± 2.53%, respectively. For group 3, a creatinine clearance rate of 93.21 ± 3.19% has been achieved, whereas the BSA rejection rate was 9.82 ± 1.47%. The comparison of creatinine clearances and BSA rejections percentages in Figure 5d exhibits that both NT-based membranes show over 90% of creatinine clearance. The drop in creatinine clearance at group 3 can be caused because of the increase in tortuosity and decrease in porosity by the nanomorphology shift from aligned NTs to chaotic NWs.^78^ Due to the negative charge of BSA molecules at pH 7, the negatively charged TiF_6_^2–^ moiety at open-NT ends repulses some of the molecules, although they are small enough to pass through the NT-based membrane’s pores.^74,79−82^ At physiological conditions, the electrostatic negative repulsive forces between the membrane surface and negatively charged blood proteins such as HSA and BSA provide the membrane anti-fouling behavior, and even a complete rejection could not be observed.^72,83^ Because of the surface hydrophilicity difference between group 3 and group 2, pure water fluxes and BSA rejections were changed. The higher BSA rejection in group 2 compared to group 3 can be explained by the fact that the repulsive force was weakened by attractive forces causing absorption of BSA molecules on the TiO_2_ NW network because of the relative hydrophobicity of amorphous NWs and a higher surface area.^84−86^ Although both NT-based membranes reject a part of albumin, the BSA rejection performances need to be increased to the acceptable limit of 70% for preventing albumin loss.^87^

**Figure 6:**
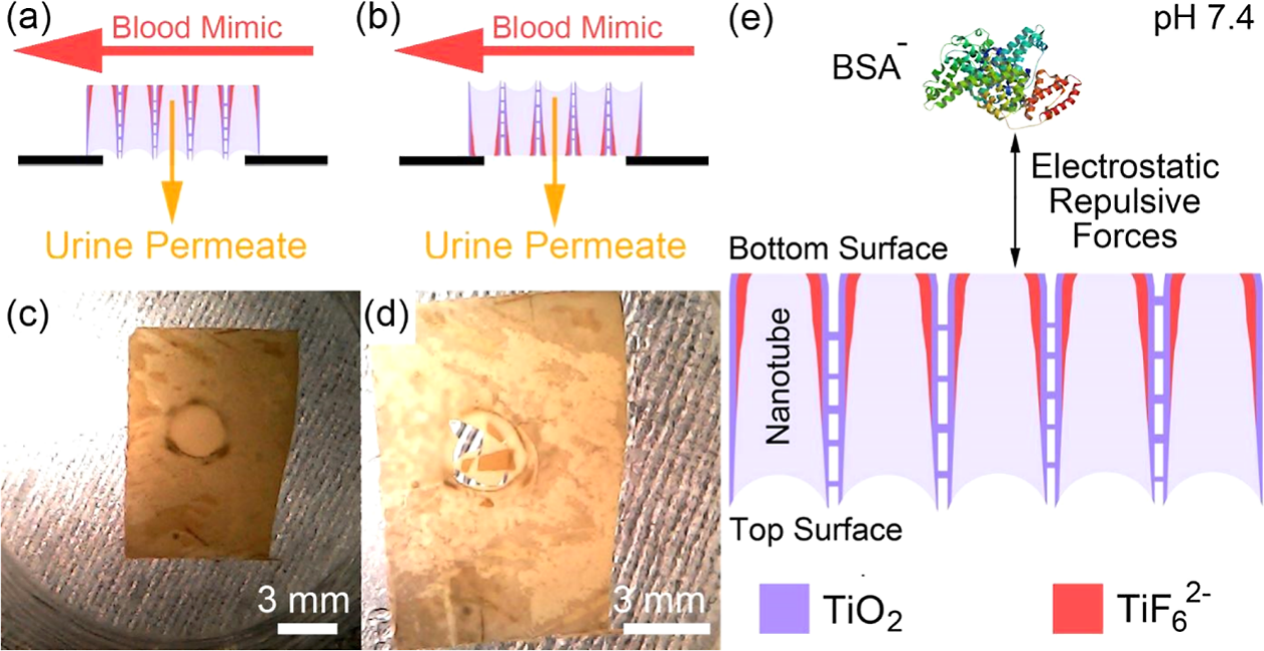
Schematic expression of liquid/solid interaction cross-flows: mechanically stable (a) and mechanically failed (b); optical images of the mechanically failed membrane: before (c) and after (d) experiments; and schematic expression of electrostatic repulsive force between the negatively charged BSA molecule and the TiF_6_^2–^-containing membrane surface at pH 7.4 (e).

**Figure 7:**
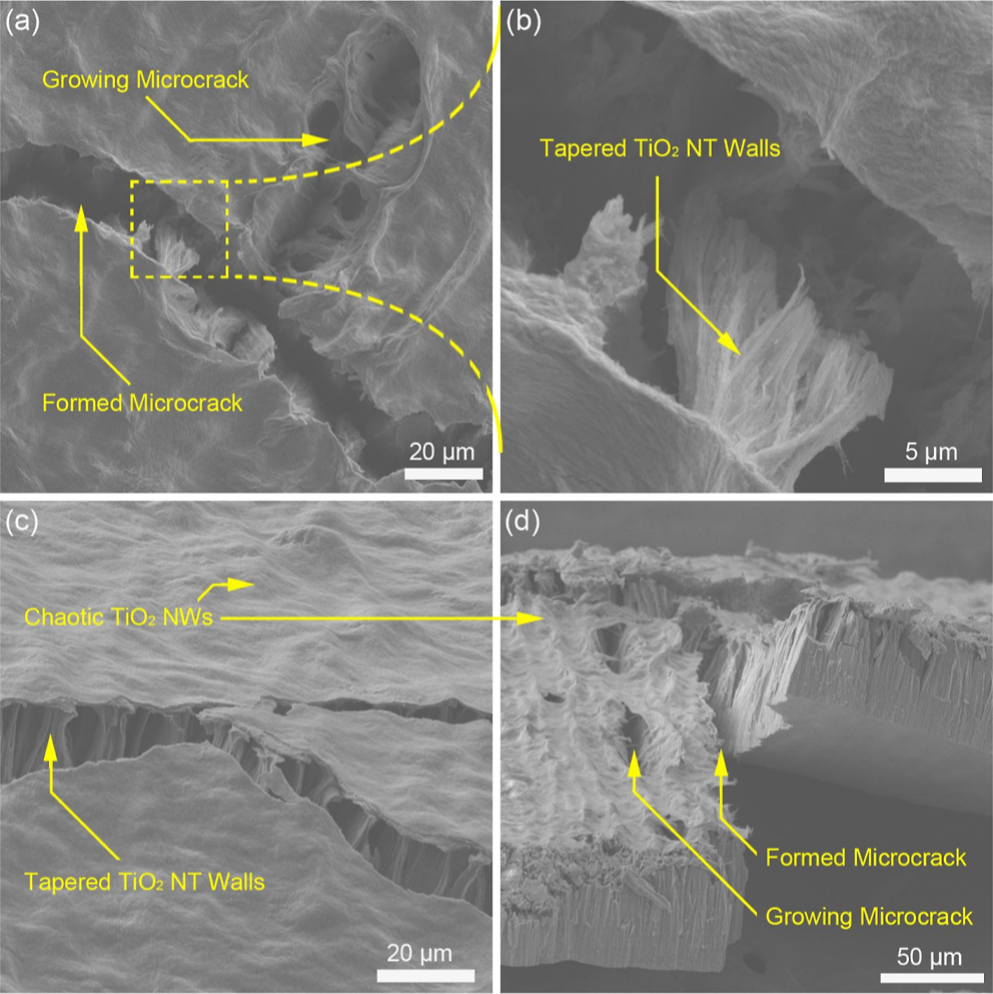
SEM images of mechanically failed group 2 membrane: topographical view of growing and formed microcracks (a), microcrack forming tapered NT walls (b), tilted image of the top surface with tapered NT walls underneath chaotic NWs (c), and cross-sectional view of the fracture point in the sample (d).

Group 2 and group 3 found were tested in 3D printed IAK models as previously described in the cross-flow of blood mimicking solution. Due to tapered and bundled NT walls and microcrack-like narrow valleys, the top surfaces consisting of aligned NTs and/or chaotic NWs were more fragile than the bottom surfaces consisting of open-end NTs in the liquid/solid surface interaction during cross-flow of blood mimicking and fouling solutions. Under the cross-flow where the liquid/solid surface interacts, as shown in Figure 6a, both group 2 and group 3 samples sustain their mechanical stability over 24 h. However, under the cross-flow where the liquid/solid surface interacts, as shown in Figure 6b, all samples mechanically failed with macrofractures as in Figure 6d after the first 10 min of process although placed without any damage as in Figure 6c. After membrane failure, extreme over-flow and molecular concentration instability were observed in collected samples. For a mechanically failed group 2 membrane, detailed topographical, tilted, and cross-sectional SEM investigation is presented in Figure 7, and regions of growing microcracks, tapered NT walls, and chaotic nanofiber formations are indicated. As mentioned before, the roughness value of these surfaces was below the acceptable hemolysis thresholds, but the increasing roughness and surface complexity caused membrane crack formation and growth as the flow continued on the surface. Another point that must be taken into account is that the chaotic NWs, which are the artifacts of highly tapered NT walls, might move freely in the aqueous environment with the cross-flow, increase the friction with high surface area per unit volume, and trigger the crack propagation with increased pressure along the newly formed cracks.

## Conclusions

Titania NT-based membranes with different surface nanomorphologies were fabricated by multi-step anodic oxidation. Characterizations and membrane performance tests showed that surface nanomorphology directly affects the bioapplication requirements. Impermeability of close-ended NTs showed that the mass transport through NT-based membranes only occurs from inside of the NTs, not through the nanobamboo structure holding neighboring NTs together. When pure water permeabilities of open-end NT membranes were compared, the top surface consisting of aligned NTs showed higher water permeability than the chaotic NWs. The tapered NT walls make the nanomembrane mechanically less stable at cross-flow of blood mimicking solution by the valley-like microcracks on the aligned NT on the top surface. The bottom surface consisted of open-ended NTs presented mechanical stability during the interaction with the flow of blood mimicking solution. When the morphology of the back surface of the membrane shifted from chaotic NWs to aligned NTs, an increase in pure water flux, creatinine clearance, and BSA rejection was observed due to hydrophilicity increase. The surface roughness of the biocompatible and superhydrophilic bottom surface interacting with the blood flow is safe enough to prevent hemolysis for both membranes. Free-standing membranes were determined to have antifouling features because of electrostatic negative repulsive forces between TiF_6_^2–^-rich regions and BSA at physiological alkalinity. The electrostatic interaction between the fabrication residual at the bottom surface and negatively charged biomolecules in the blood mimicking solution caused a small amount of rejection. For both membranes, although creatinine clearance is acceptable to being over 90%, normalized flux is acceptable to being stable at 50% after 24 h, and urine output flux can be optimized by scaling of the effective surface area of the membrane, and an improvement in BSA rejection performance is extremely necessary from near 20% to above 70%. We strongly predict that the membrane surface can be chemically decorated with polyanionic or zwitterionic functional modifications to improve charged protein rejection. The electrochemically fabricated membranes discussed in the article are currently limited in their suitability for miniaturized applications. However, due to their scalable and low-cost fabrication, biocompatibility, low surface roughness, high water permeability, and high surface wettability, titanium NT-based membranes are promising nanoceramic membranes which can be enhanced in IAK applications with the suggested improvements.

## Supporting Information Available

Schematic expression of sudden electrode transfer, graph of simultaneous current–voltage–temperature change during post-anodization of group 2 samples, schematic expression of the cross-flow test mechanism used in membrane performance tests, SEM images of microcracks (topographical and cross-sectional) and nanobamboo structures observed with fabricated titania NT-based membranes, SEM image of the surface where EDX analysis is carried out, EDX spectra of spectrum 9 and spectrum 13, and AFM investigation of top surfaces of group 1–3 and group 2 samples (PDF)
