# Supplementary material for: Electrostatic Repulsive Features of Free-Standing Titanium Dioxide Nanotube-Based Membranes in Biofiltration Applications
Source: Langmuir. 2023 Feb 14;39(9):3400–10. doi: 10.1021/acs.langmuir.2c03331 (PMC9996822; doi:10.1021/acs.langmuir.2c03331)
Supplement: Supplementary file 1 — la2c03331_si_001.pdf [file la2c03331_si_001.pdf]

SUPPORTING INFORMATION

Electrostatic Repulsive Features of Free-standing Titanium Dioxide Nanotube-based Membranes in Biofiltration Applications

Bogac Kilicarslan[1], Melis Sardan Ekiz[2], Cem Bayram[1]*

Department of Nanotechnology and Nanomedicine, Graduate School of Science and Engineering, Hacettepe University, Ankara, 06800, Turkey[1]

Advanced Technologies Application and Research Centre, Hacettepe University, Ankara, 06800, Turkey[2]

Table of Contents

1. Schematic expression of sudden electrode transfer, and graph of simultaneous current-voltage-temperature change during post-anodization of Group 2 samples.
2. Schematic expression of cross flow test mechanism used in membrane performance tests.
3. SEM images of microcracks (topographical and crossectional) and nanobamboo structures observed with fabricated titania nanotube-based membranes.
4. SEM image of the surface where EDX analysis done, and EDX spectra of spectrum 9 and spectrum 13.
5. AFM investigation of top surfaces of Group 1-3 and Group 2 samples.

Corresponding Author

Cem Bayram – Graduate School of Science and Engineering, Department of Nanotechnology and Nanomedicine, Hacettepe University, Ankara, Turkey; orcid.org/0000-0001-8717-4668; E-mail: cemb@hacettepe.edu.tr

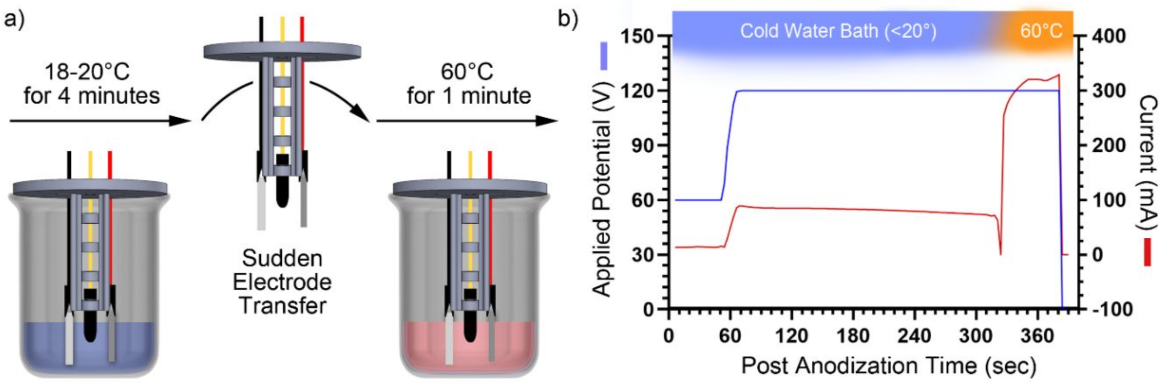

Figure S1. Schematic expression of sudden electrode transfer (a), and graph of simultaneous current-voltage-temperature change during post-anodization of Group 2 samples (b).

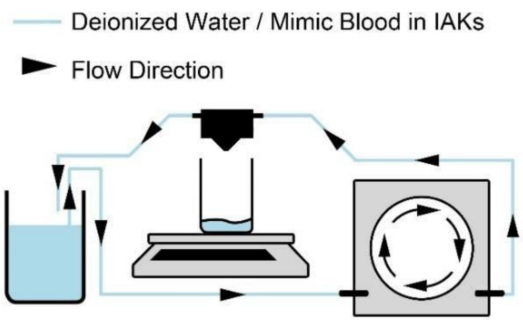

Figure S2. Schematic expression of cross flow test mechanism used in membrane performance tests.

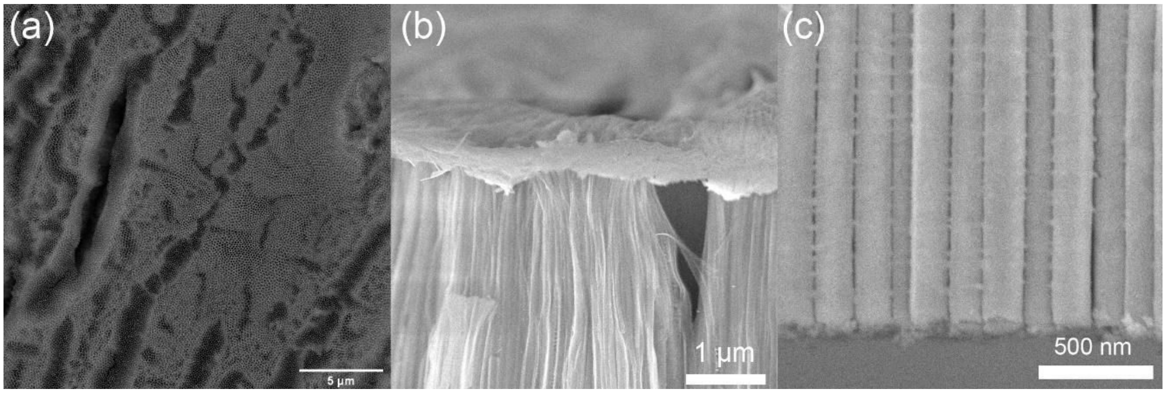

Figure S3. SEM images of microcracks: topographical (a) and crossectional (b) and nanobamboo structures observed with fabricated titania nanotube-based membranes (c).

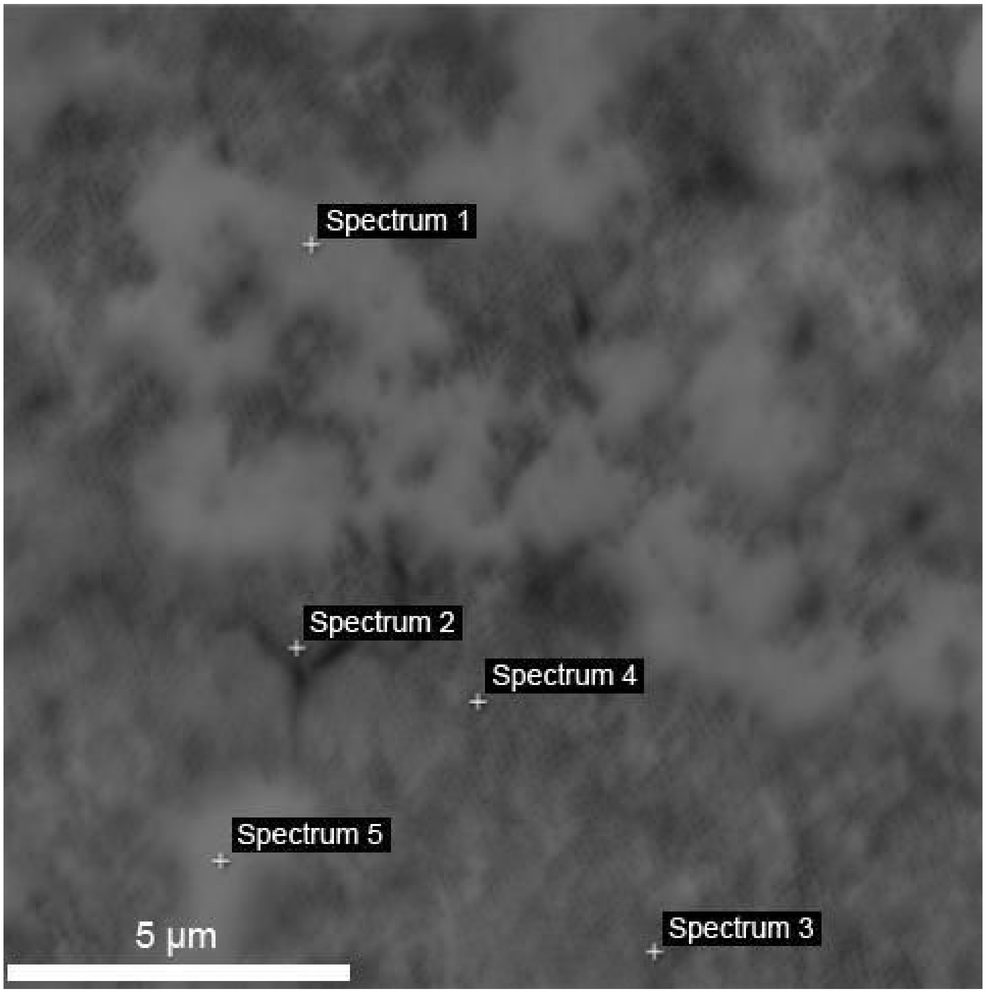

Figure S4. SEM image of investigation zone during EDX analysis at the bottom surface (open-end NTs) of membrane.

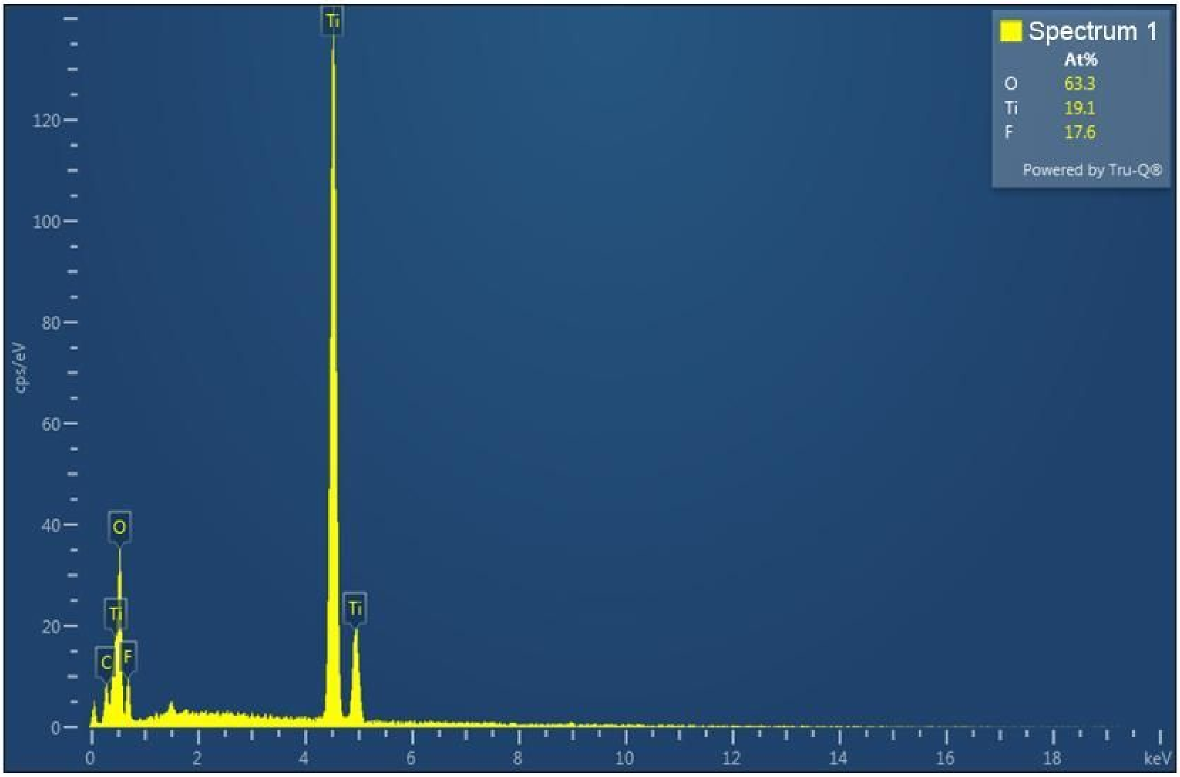

Figure S5. EDX spectra at spectrum 1.

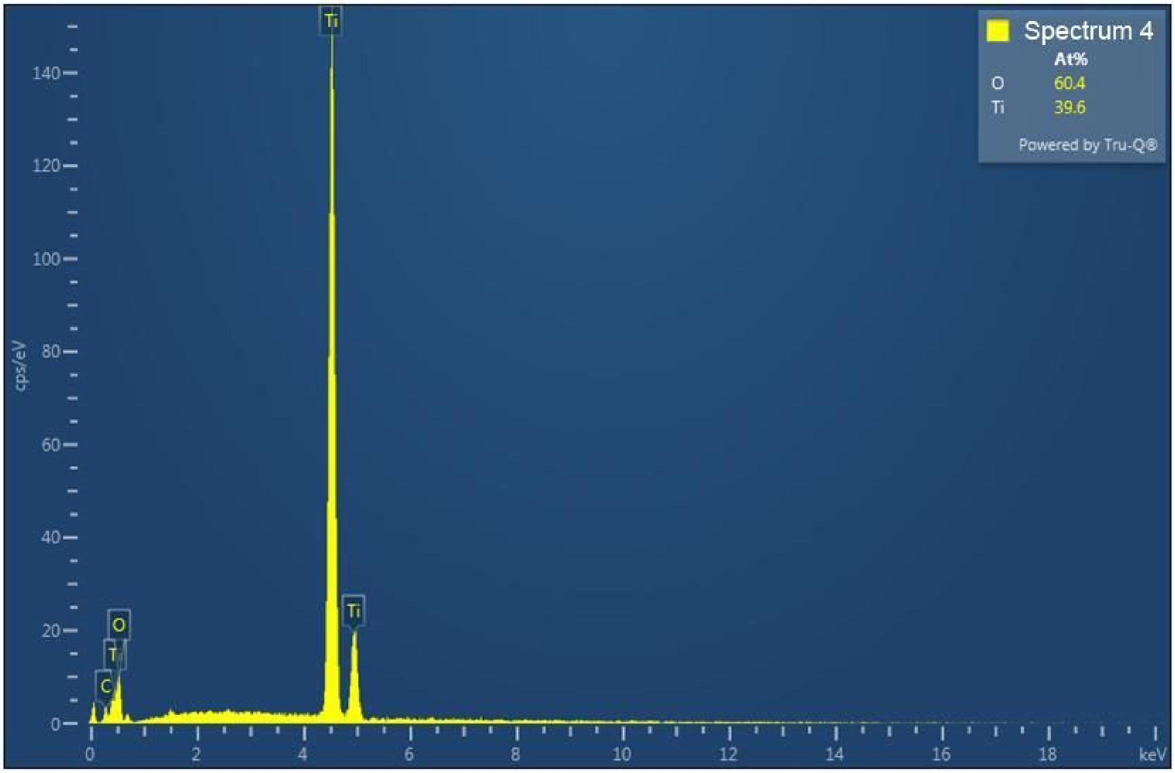

Figure S6. EDX spectra at spectrum 4.

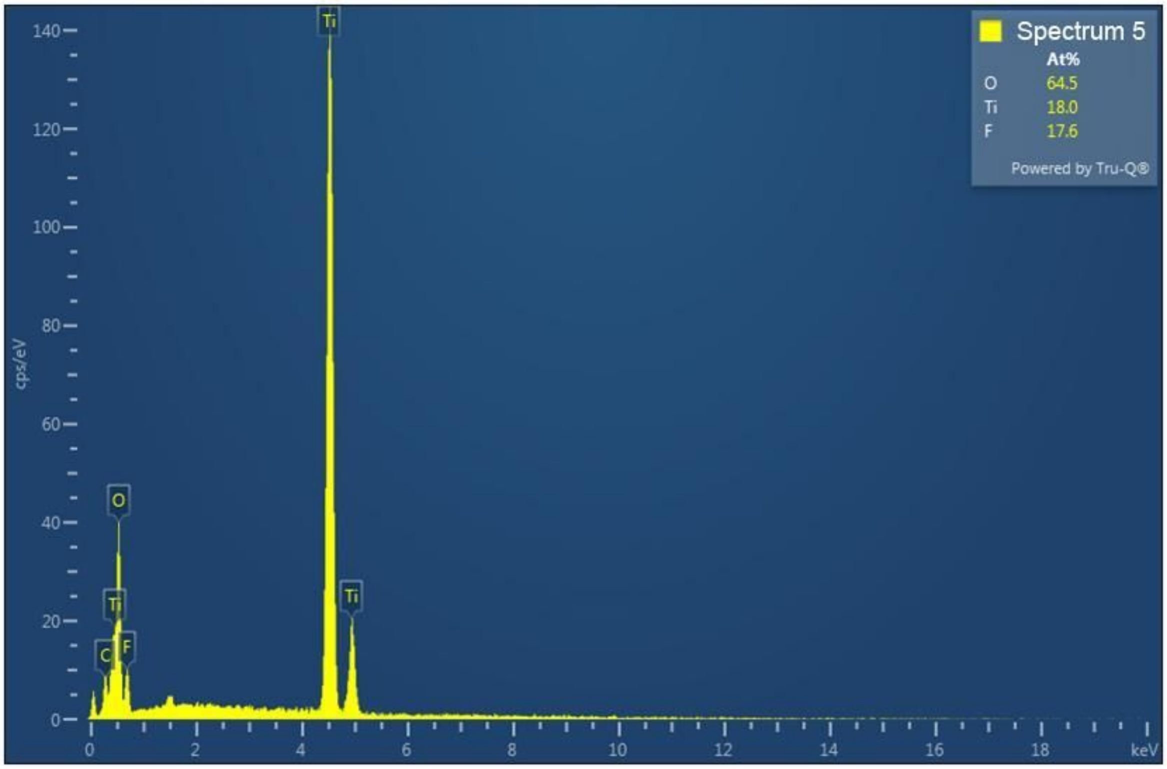

Figure S7. EDX spectra at spectrum 5.

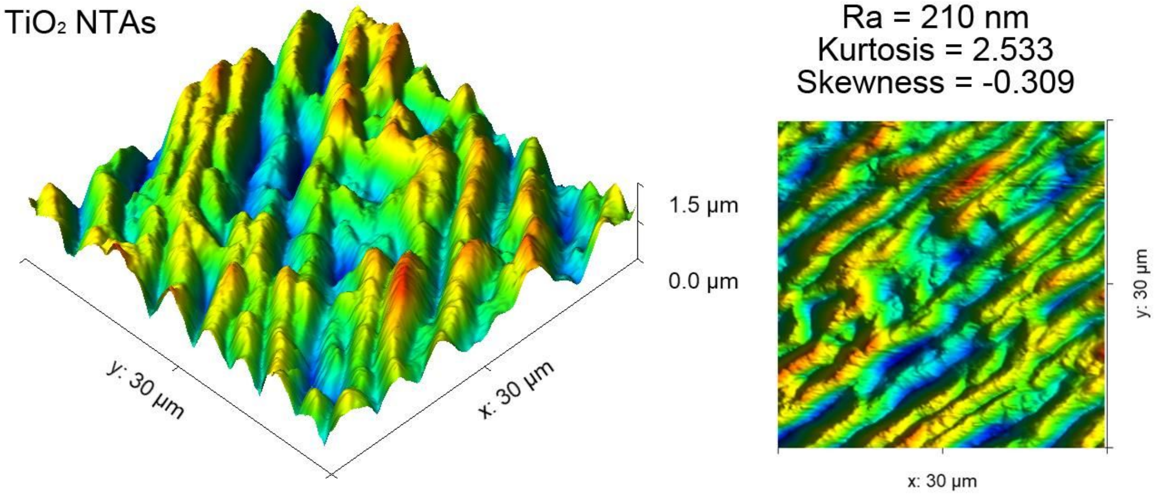

Figure S8. Surface roughness (AFM) investigation at the top surface of Group 1 and 3 samples.

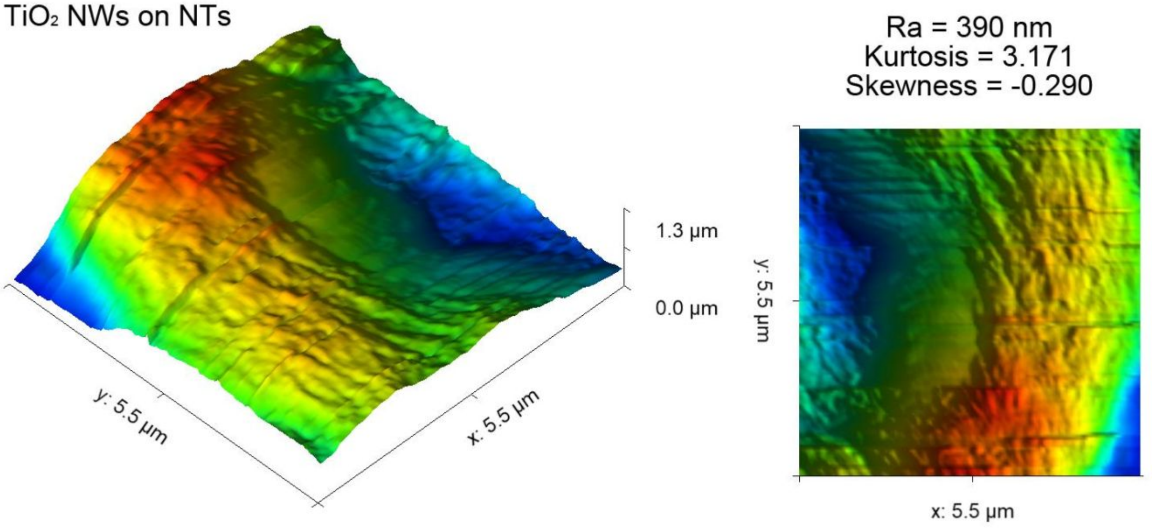

Figure S9. Surface roughness (AFM) investigation at the top surface of Group 2 samples.
